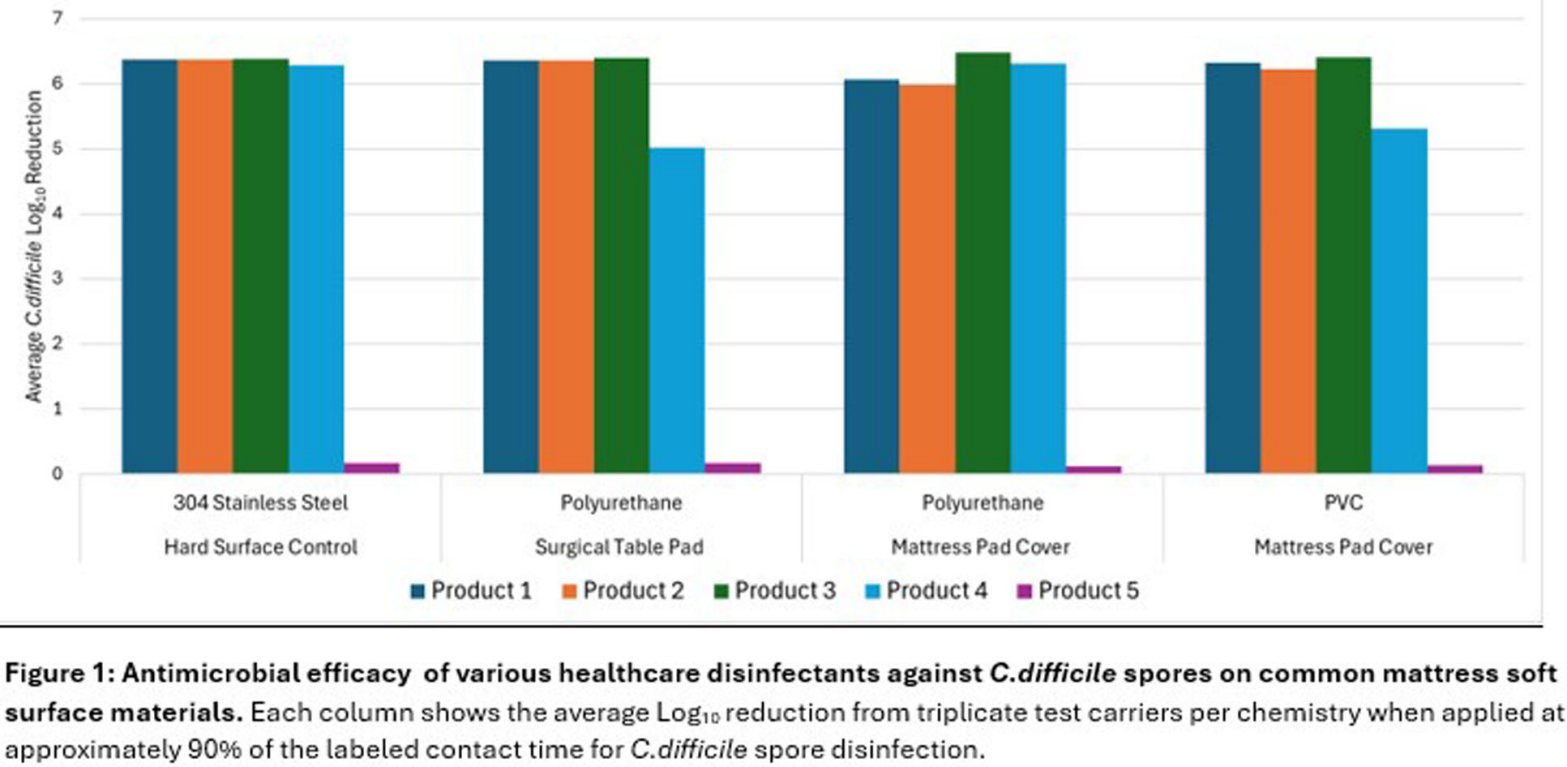# 216 Epidemiology of Carbapenemase-Producing Organisms (CPOs) — San Francisco, 2023-2025

**DOI:** 10.1017/ash.2026.10599

**Published:** 2026-06-23

**Authors:** Joshua Luedtke, Teresa Podtburg, Laura Willson, Kris Owens, Karoline Sperling, Don Rotter, Joseph Wegner

**Affiliations:** 1 Ecolab

## Abstract

**Background:** Clostridioides difficile is a spore-forming bacterium that causes severe healthcare-associated infections, especially in immunocompromised patients and those exposed to antibiotics. C.difficile spores can persist in the environment, making routine use of sporicidal disinfectants essential for reducing contamination and infection risk. Surgical and patient mattresses, while impervious, are soft and flexible and generally do not meet the U.S Environmental Protection Agency (EPA) definition of a hard, nonporous surface, which is the surface type typically used to test disinfectant efficacy. Assessing spore survival and disinfectant performance on these soft surfaces is important for minimizing transmission risk, especially since they are often treated like hard surfaces. **Methods:** Antimicrobial efficacy testing was conducted using common healthcare disinfectants against C.difficile, following standard operating procedures typically required for EPA registration. Spores were inoculated and dried onto stainless steel coupons (hard surface control) and multiple soft surface coupons made of common mattress cover materials. Each disinfectant formulation represented a common active ingredient or blend of active ingredients, ranging from ready-to-use sprays to wipes. Products were tested at approximately 90% of the manufacturer-defined contact times to stress the chemistry and reveal any efficacy differences between the different surface types. **Results:** Significant C.difficile Log reductions were observed with disinfectant Products 1, 2, 3, and 4, demonstrating high efficacy against C.difficile. Product 5, a non-sporicidal disinfectant commonly used on mattress covers, demonstrated little to no reduction of C.difficile. **Conclusion:** Using standard benchtop testing methods, this study aimed to understand the efficacy of sporicidal healthcare disinfectants against C.difficile on common soft surface materials. While EPA-registered disinfectants may be intended for hard, nonporous surfaces, the use of sporicidal disinfectants on mattresses may be more effective at reducing the risk of infection than traditionally thought, especially since these soft surfaces cannot be laundered. This is particularly important during outbreaks and underscores the proactive use of sporicidal disinfectants to mitigate C.difficile and other emerging pathogens.

**Figure:**